# Hunter on Slayton

**Published:** 1859-12

**Authors:** Wm. M. Hunter


### HUNTER ON SLAYTON.

MESSRS EDITORS :—I resume my pencil (I abhor ink, "the black and midnight hag," it stains my fingers,) in answer to Dr. Slayton's proposition in the November No. of the Register. It was Job, I believe, who exclaimed, "Oh that mine adversary had written a book." Slayton has written enough to fill a small sized primer, principally devoted to a certain improvement (?) professional ethics, how to obtain a patent surreptitiously, and the game of brag; after reading which, I can fully understand Job's philosophy. In his last instalment, he commences with finding fault with my language, thereby hoping to gain sympathy for his cause. I have only to say, that I am disposed to call things by their right names, and don't believe in mistifying truth in clouded language, when good old Saxon is clear and to the point. Hence, when a man deliberately writes that concerning myself which is untrue, and I know that he is aware of it, I pronounce it a lie, as there is no other word for it without circumlocution; and I do not know by what code of ethics I should be debarred from telling the truth.

In the July No., I stated that Slayton had applied for a patent, and that when issued it would be worth "just precisely its bulk in blank paper;" and now we have his own testimony to that effect; for, to quote his own words in his last, he says: — "In my specifications I simply state that I use gold or silver *properly* prepared, leaving just enough untold to defeat just such efforts as you are now making." Be it so; then his process is not a *patented* one, but a *secret*; and he has, in direct violation of the law, and premeditatedly, as the above quotation shows, surreptitiously obtained a patent. Here is the law:

"But before any inventor shall receive a patent for any such new invention or discovery, he shall deliver a written description of his invention or discovery, and of the manner and process of making, constructing, using and compounding the same, in such full, clear, and exact terms, avoiding unnecessary prolixity, as to enable any person skilled in the art or science to which it appertains, or with which it is most nearly connected, to make, construct, compound and use the same."

"*And be it further enacted,* That, whenever any patent, which has heretofore been granted, or which shall hereafter be granted, shall be inoperative or invalid, by reason of a defective or insufficient description or specification, or by reason of the patentee claiming in his specifications as his own invention, more than he had or shall have a right to claim as new, if the error has, or shall have arisen by inadvertency, accident, or mistake, and without any *fraudulent or deceptive intention,* it shall be lawful for the commissioner, upon surrender to him of such patent, and the payment of the further duty of fifteen dollars, to cause a new patent to be issued to the said inventor for the same invention, for the residue of the period then unexpired, for which the original patent was granted, in accordance with the patentee's corrected description and specification."

And yet, in defiance of this, he boasts that the soul of the thing is a *secret,* (will this dodge sell it?) and, I may further add, that if his work is like that exhibited in Cincinnati, a secret not worth knowing.

Let us review the subject. In the first place, he makes application under the law for a patent, and, is required by law to give a full, clear and exact description, &c.; and furthermore, to *swear* that he is the original inventor of the art for which he solicits a patent, which he has or should have done, he obtains a patent, and coolly informs those persons who have a right to the process after his monopoly has expired, which monopoly is granted him for the faithful observance of the law in its requirements, it cannot be done according to my specifications. I have purposely withheld that which is necessary to success; or, in other words, I have used letters patent as a decoy duck to sell you a secret. Very sharp practice if it was worth anything; but he has overreached himself; for, even were it a good thing, the second section of the law quoted would prevent him from getting a *valid* patent, were it of sufficient importance to oppose; he having acknowledged that it was not through inadvertency, accident, or mistake, that the specification is defective; but from design. In his brag, he labors hard to get himself before the profession on certain terms, which he himself makes; for instance, to test the strength of his work, and "*neither piece to be marred or injured in any way*," and by ruling out the very work that I brought in opposition to it, viz: tin. See Dental Register, July No., page 25, objection 3; and, in the second place, to have it placed beside *ordinary* gold work single gum teeth, I presume taken from the mouth of a patient, who is to go without their teeth for a week, to prove what every intelligent dentist knows, that, fitted ever so carefully, the piece will be in just the same condition, with the so-called improvement; having admitted the fluids of the mouth in the interstices from whence ordinary cleaning would not remove the putrid and offensive matter, "presenting a beautiful appearance of cleanliness, but in reality, filthy beyond description." Very cute, in our very cute brother Slayton, but the brag won't do. I would not accept the proposition, even were I *allowed* to present such work as I know is comparatively free from the objection named, for the simple reason that a gentleman never accepts a proposition accompanied by a threat.

WM. M. HUNTER.

